# IgE Plasma Cell Leukemia Harboring t(11;14) and 1q Amplification

**DOI:** 10.1155/2023/4747989

**Published:** 2023-06-27

**Authors:** Wataru Nakahara, Takahito Ogawa, Hitomi Matsunaga, Yuki Iwasa, Momoka Horita, Mako Ikeda, Mizuki Asako, Sadaharu Iio, Yuki Iwama, Kazumasa Oka, Shuji Ueda

**Affiliations:** ^1^Department of Hematology, Hyogo Prefectural Nishinomiya Hospital, Nishinomiya, Hyogo, Japan; ^2^Department of Clinical Laboratory, Hyogo Prefectural Nishinomiya Hospital, Nishinomiya, Hyogo, Japan; ^3^Department of Gastroenterology, Hyogo Prefectural Nishinomiya Hospital, Nishinomiya, Hyogo, Japan; ^4^Department of Radiology, Hyogo Prefectural Nishinomiya Hospital, Nishinomiya, Hyogo, Japan; ^5^Department of Pathology, Hyogo Prefectural Nishinomiya Hospital, Nishinomiya, Hyogo, Japan

## Abstract

IgE plasma cell neoplasm is the rarest subtype of plasma cell neoplasms and is known for its poor prognosis and high incidence of t(11;14). However, t(11;14) has been classified as a standard-risk rather than high-risk cytogenetic abnormality in multiple myeloma. We have been unable to explain the discrepancy that the hallmark of IgE plasma cell neoplasm with a poor prognosis is a standard-risk cytogenetic abnormality. Here, we report a case of IgE primary plasma cell leukemia with extramedullary lesions of the liver, stomach, and lymph nodes. Plasma cell infiltration was pathologically confirmed in each organ. Cytogenetic analysis of plasma cells revealed t(11;14) and amplification of 1q21. Chemotherapy, with immunomodulatory imide drugs, proteasome inhibitors, and CD38 antibodies, was unsuccessful. In IgE plasma cell neoplasm, coexistence of other cytogenetic abnormalities with t(11;14) may be important. Investigating the presence of cytogenetic abnormalities coexisting with t(11;14) is not only useful for evaluating prognosis but also important for understanding the pathogenesis of the disease. Recently, venetoclax, an oral BCL2 inhibitor, has demonstrated promising efficacy in plasma cell neoplasm patients harboring t(11;14). Development of an effective venetoclax-based regimen for treating aggressive IgE plasma cell neoplasm with t(11;14) is expected.

## 1. Introduction

Plasma cell neoplasms, including monoclonal gammopathy of undetermined significance (MGUS), multiple myeloma (MM), and plasma cell leukemia (PCL), are characterized by clonal proliferation of malignant plasma cells in the bone marrow leading to overproduction of monoclonal immunoglobulins. IgE plasma cell neoplasm is the rarest subtype of plasma cell neoplasms [1]. Compared with other immunoglobulin subtypes, IgE plasma cell neoplasm shows a more aggressive clinical course and has unique clinical features, i.e., a higher incidence of PCL and the presence of t(11;14) [2, 3].

Here, we report on a patient with IgE primary PCL with multiple organ involvement of the liver, stomach, and lymph nodes showing the cytogenetic abnormality of 1q21 amplification in addition to t(11;14).

## 2. Case Presentation

A 63-year-old Japanese man was admitted to our hospital for evaluation of monoclonal proteinemia. Three weeks before this hospitalization, he was admitted to another hospital because of recent diarrhea and anorexia, with weight loss of 10 kg in the preceding 2 months. A peripheral blood analysis showed anemia and thrombocytopenia. Biochemical analysis revealed impairment of liver and renal function, and serum protein electrophoresis revealed a monoclonal protein. MM was suspected, and after administration of bisphosphonate to improve mild hypercalcemia (11.1 mg/dL), the patient was transferred to our hospital.

**Figure 1:**
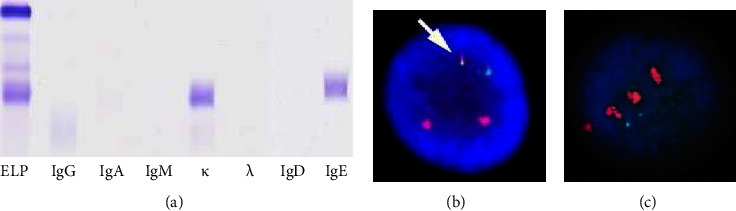
(a) Serum immunofixation demonstrating a monoclonal IgE kappa (*κ*) component, (b) interphase fluorescence in situ hybridization (FISH) with dual fusion probes for immunoglobulin heavy chain (IGH) and cyclin D1 (CCND1) showed a fusion signal corresponding to t(11;14)/IGH-CCND1 fusion (white arrow), and (c) interphase FISH with a probe for cyclin-dependent kinases regulatory subunit 1 (CKS1B), a chromosome 1q marker, showed four copies of CKS1B. ELP: serum protein electrophoresis.

**Figure 2:**
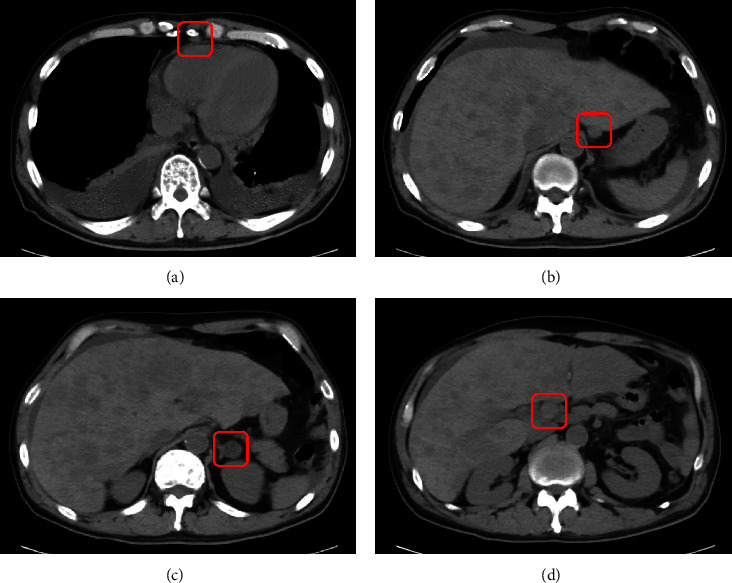
Computed tomography revealed bilateral pleural effusion (a), multiple hypodense liver nodules (b–d), thickening of the stomach wall (b), ascites (b–d), and swelling of multiple lymph nodes (red boxes in (a–d)).

**Figure 3:**
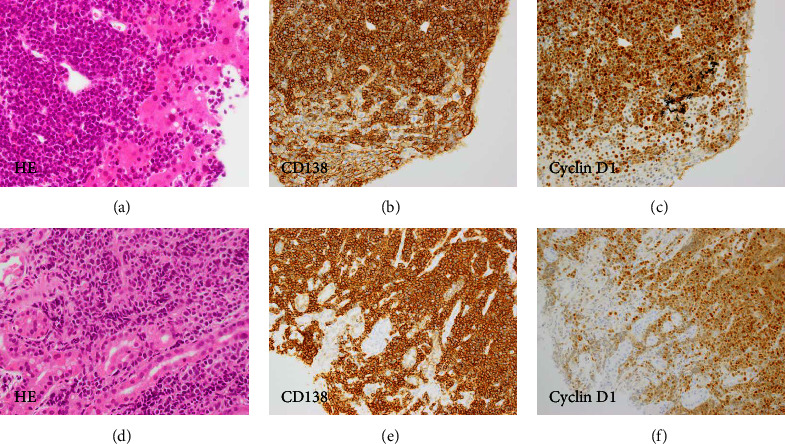
Histopathological examination of biopsy samples from the liver and gastric mucosa. The plasma cells had diffusely infiltrated the liver (a–c) and gastric mucosa (d–f) (hematoxylin-eosin staining, ×400). The plasma cells were positive for CD138 (b and e) and cyclin D1 (c and f) (×200). HE: hematoxylin-eosin staining.

On physical examination, the liver was palpable four fingerbreadths below the right costal margin and edema was observed in both lower legs. A peripheral blood count showed a white blood cell count of 4.9 × 10^9^/L, a hemoglobin level of 7.9 g/dL, and a platelet count of 34 × 10^9^/L. Morphologic analysis of the peripheral blood showed 5% plasma cells with leukoerythroblastosis. A biochemical examination revealed liver dysfunction and an elevated serum creatinine level (Table 1). The serum calcium level had been normalized by the administration of bisphosphonate. The serum IgE concentration was high (4,720,000 IU/mL, which is equivalent to 1,133 mg/dL). Serum protein electrophoresis and immunofixation electrophoresis confirmed the presence of IgE kappa (*κ*) paraprotein (Figure 1(a)). The laboratory values on admission and corresponding reference ranges are shown in the Table 1. Bone marrow aspirate revealed 68.6% plasma cells which were positive for CD56 and *κ* and negative for CD20 in the flow cytometry analysis. Immunohistochemistry of the bone marrow biopsy showed plasma cells positive for CD138, *κ*, and cyclin D1 (CCND1). Cytogenetic analysis of bone marrow cells showed a complex karyotype that included t(11;14) (q13; q32), which was indicative of (CCND1)/immunoglobulin heavy chain (IGH) fusion. Results of the fluorescence in situ hybridization (FISH) were negative for the translocations of FGFR3 and MAF and the deletion of 17p13 and 13q14 but positive for the CCND1/IGH fusion and the amplification (four copies) of 1q21, including CKS1B gene (Figures 1(b) and 1(c)). The patient was diagnosed with IgE *κ* type primary PCL according to the revised diagnostic criteria of the International Myeloma Working Group (IMWG) [4]. A systemic examination with computed tomography (CT) confirmed bilateral pleural effusion, ascites, multiple hypodense nodules in the liver, thickening of the stomach wall, and multiple lymph node swellings (Figure 2). Needle biopsy of a liver nodule showed a diffuse infiltration of plasma cells, which were positive for CD138 and CCND1 (Figure 3). A gastrointestinal endoscopy performed to examine the cause of the appetite loss revealed multiple gastric ulcers. Pathological examination of the gastric mucosa biopsy revealed CD138-positive plasma cell infiltration (Figure 3).

We started a DVd regimen consisting of daratumumab (16 mg/kg/week), bortezomib (1.3 mg/m^2^ twice a week), and dexamethasone (20 mg/body/week). However, DVd treatment was ineffective and did not control disease progression. Ten days after starting DVd treatment, we changed to an IsaPd regimen with isatuximab (10 mg/kg/week), pomalidomide (4 mg/day), and dexamethasone (20 mg/body/week); however, the patient died of the disease two days after the start of IsaPd treatment. With the consent of the patient's family, an autopsy was performed after his death. Extensive plasma cell proliferation and invasion were confirmed in the bone marrow, stomach, liver, and lymph nodes.

## 3. Discussion

PCL is a rare, yet aggressive form of MM characterized by plasma cells circulating in the peripheral blood. The original diagnostic criteria of PCL established by Kyle in 1974 required both more than 20% circulating plasma cells (CPCs) and an absolute count greater than 2 × 10^9^/L plasma cells in peripheral blood, but the IMWG has recently revised the diagnostic criteria for PCL to a lower cut-off value of 5% CPCs in the peripheral blood [4, 5]. This revision is based on two recent retrospective studies which demonstrated that the percentage of ≥5% CPCs in patients with MM had a similar adverse prognostic impact as the previously defined PCL [6, 7]. The aggressive clinical course of our patient who had only 5% CPCs is consistent with these findings.

IgE plasma cell neoplasm is the rarest subtype of plasma cell neoplasm and accounts for only 0.01% of all cases; insights into IgE plasma cell neoplasm are limited by its rarity [1]. Although the clinical features of IgE plasma cell neoplasm are generally similar to those of other immunoglobulin subtypes, IgE plasma cell neoplasm is characterized in particular by a high frequency of PCL and the presence of t(11;14) (q13; q32) [2, 3, 8]. However, the frequency of PCL and t(11;14) in the IgE plasma cell neoplasm has not been well defined. To the best of our knowledge, fewer than 80 cases of IgE plasma cell neoplasm have been reported since the first case was published in 1967. In 2017, Hejl et al. reviewed the 63 previously published cases of IgE monoclonal gammopathy, which comprised cases of MM, PCL, transient M proteinemia, and IgE monoclonal gammopathy associated with other diseases [9]. We re-reviewed 74 previously published cases, i.e., the 63 cases reviewed by Hejl et al. in 2017, 5 cases that were missing in the review by Hejl et al., and 6 cases that have been reported since the publication by Hejl et al. [9–18]. Of these 74 cases, 51 were MM, 11 were PCL (7 of which were primary and 4 of which were secondary), 5 were monoclonal gammopathy of undetermined significance, and 7 were monoclonal gammopathy associated with other diseases. Cytogenetic examinations, such as chromosomal karyotyping with G-banding and FISH analysis, were not available for cases reported before 1994. Of the 62 cases of MM and PCL, chromosomal karyotyping or FISH analysis was performed in 16 cases and showed that 8 of these 16 cases (50%) had either t(11;14) (q13; q32), as shown by chromosomal karyotyping, or CCND1 translocation, as shown by FISH analysis. The CCND1 translocation was shown by FISH analysis in 6 out of 9 cases (66%), supporting previous reports that CCND1 translocation may be the hallmark of IgE plasma cell neoplasm.

Both concomitant and subsequent extramedullary involvement of liver, spleen, lymph nodes, and other soft tissues are common in PCL, and multiple extramedullary lesions such as the liver, stomach, and lymph nodes were confirmed in our patient as well [19]. In PCL as well as in IgE plasma cell neoplasm, the most common IGH translocation is t(11;14), which is seen in 49% to 65% of cases, highlighting the importance of this rearrangement in the etiology of PCL [20]. On the other hand, although t(4;14), t(14;16), t(14;20), del (17p), nonhyperdiploidy, and 1q gain were identified as high-risk cytogenetic abnormalities, t(11; 14) is classified as a standard-risk according to the International Myeloma Working Group guidelines [21]. The discrepancy that the hallmark of IgE plasma cell neoplasm and PCL which have been known to be poor prognosis is a standard-risk cytogenetic abnormality remains to be elucidated. A possible reason for the poor prognosis of IgE plasma cell neoplasm and PCL with t(11;14) is the coexistence of other cytogenetic abnormalities with t(11;14). Leiba et al. examined the cooccurrence rates of t(11;14) with seven cytogenetic aberrations, i.e., del (13q), del (17p), del (1p), gain (1q), 1q amplification, del (16q), and del (IGH), in 212 patients newly diagnosed with MM who carried t(11;14) [22]. Their study demonstrated that 60% of patients had at least 1 cytogenetic abnormality in addition to t(11;14), indicating a high rate of coexistence of t(11;14) with additional cytogenetic abnormalities. The presence of t(11;14) combined with other aberrations, especially 1q amplification, del (1p), and del (IGH), had a profoundly deleterious effect on the overall survival of patients compared with patients with t(11;14) only [22]. Of great interest in our case is that we found 1q amplification in addition to t(11;14); 1q21 gain/amplification was recently identified as a genetic driver of high-risk MM and has been reported to be frequently observed in both PCL (especially primary PCL) and extramedullary MM [23, 24]. Galakhoff et al. reported a case of IgE MM that had transformed to PCL with the gain of 1q21 (3 copies) and IGH rearrangement, and this genetic abnormality may be a high malignant potential factor in IgE plasma cell neoplasm [17]. In IgE plasma cell neoplasm with a high frequency of t(11;14), investigating the presence of cytogenetic abnormalities coexisting with t(11;14) is not only useful for evaluating a patient's prognosis but also important for understanding the pathogenesis of IgE plasma cell neoplasm. To date, FISH analysis has been performed in nine cases of IgE plasma cell neoplasm and the cytogenetic abnormalities have been reported; however, in most cases, cytogenetic abnormalities other than IGH translocations have not been examined. In IgE plasma cell neoplasm, further investigation of the cytogenetic abnormalities present in addition to t(11;14) is warranted, including the type, frequency, and relationship to the pathological condition.

Some cases of IgE plasma cell neoplasm have been successfully treated with novel agents, such as immunomodulatory imide drugs (IMiDs) and proteasome inhibitors (PIs), and these drugs are expected to improve the prognosis of this disease [13]. However, our patient did not fully respond to treatment with an IMiD and PI, which were administered together with CD38 antibodies. To our knowledge, no reports have described the efficacy of these novel drugs such as IMiDs, PIs, and CD38 antibodies, in IgE PCL and IgE MM with extramedullary lesions [17, 25]. A new alternative therapy is needed that will improve the prognosis of patients with these highly aggressive IgE MM and PCL. Venetoclax, an oral BCL2 inhibitor, has demonstrated promising efficacy in patients with plasma cell neoplasm harboring t(11;14), which is associated with high BCL2 expression [26]. Venetoclax is included as a treatment option for relapsed/refractory t(11;14) plasma cell neoplasms in National Comprehensive Cancer Network (NCCN) guidelines, but no country has approved venetoclax for the treatment of MM, so it cannot be used in clinical practice [27]. Currently, several clinical trials of venetoclax-based combination chemotherapy for MM with t(11;14) have been completed or are underway, but unanswered questions regarding safety issues such as cytopenia-associated severe infections remains to be resolved [27]. Recently, Nalghranyan et al. reported a case of t(11;14) positive PCL with biallelic inactivation of TP53 that was treated with a reduced dose of venetoclax in combination with daratumumab and dexamethasone, resulting in stringent complete response [28]. It is hoped that after dose optimization and other modifications, venetoclax-based combination chemotherapy will be an effective treatment for PCL and aggressive IgE plasma cell neoplasm with t(11;14) and additional cytogenetic abnormalities.

## Figures and Tables

**Table 1:** Laboratory values on admission.

Blood count		Reference ranges	Biochemistry		Reference ranges
WBC, 109/L	4.9	3.3–8.6	T-bil, mg/dL	0.87	0.40–1.50
Pro, %	0.5	NA	AST, U/L	57	13–30
Mye, %	4.5	NA	ALT, U/L	123	10–42
Meta, %	5.5	NA	*γ*GTP, U/L	1547	13–64
Stab, %	8.0	0–10	LDH, U/L	287	124–222
Seg, %	33.5	28–68	CRP, mg/dL	0.16	0.00–0.14
Lym, %	34.0	17–57	BUN, mg/dL	31.4	8.0–20.0
Mono, %	6.0	33.4–44.9	Cre, mg/dL	1.76	0.65–1.07
Eos, %	1.0	0–10	UA, mg/dL	12.2	3.7–7.8
Bas, %	2.0	0–2	Na, mEq/L	136	138–145
Plasma, %	5.0	NA	K, mEq/L	4.5	3.6–4.8
Ebl, /W	13.5	NA	Cl, mEq/L	104	101–108
RBC, 1012/L	2.36	4.35–5.55	Ca, mg/dL	8.5	8.8–10.1
Hb, g/dL	7.9	13.7–16.8	TP, g/dL	6.3	6.6–8.1
Hct, %	22.8	40.7.-50.1	Alb, g/dL	3.4	4.1–5.1
Plt, 109/L	34	158–348	IgG, mg/dL	212	861–1747
Coagulation			IgA, mg/dL	30	93–393
PT, sec	14.7	10.5–13.5	IgM, mg/dL	50	33–183
PT (INR)	1.21	0.90–1.10	IgE, IU/mL	4,720,000	0–173
aPTT, sec	28.4	24.0–35.0	*β*2MG, mg/L	14.0	1.0–1.9
Fib, mg/dL	264.3	200.0–400.0	Serum-free light chain		NA
AT III, %	90.7	80.0–130.0	*κ* chain, mg/L	12.7	3.3–19.4
FDP, *μ*g/mL	2.9	<5.0	*λ* chain, mg/L	1.6	5.7–26.3
D-dimer, *μ*g/mL	0.6	<1.0	*κ*/*λ* ratio	7.94	0.26–1.65

Alb, albumin; ALT, alanine-aminotransferase; aPTT, activated partial thromboplastin time; AST, aspartate-aminotransferase; AT III, antithrombin III; Bas, basophil; BUN, blood urea nitrogen; Cre, creatinine; CRP, C reactive protein; Ebl, erythroblast; Eos, eosinophil; FDP, fibrin/fibrinogen degradation products; Fib, fibrinogen; Hb, hemoglobin; Hct, hematocrit; Ig, immunoglobulin; LDH, lactate dehydrogenase; Lym, lymphocyte; Meta, metamyelocyte; MG, macroglobulin; Mon, monocyte; Mye, myelocyte; NA, not applicable; Plasma, plasma cell; Plt, platelets; Pro, promyelocyte; PT, prothrombin time; PT (INR), prothrombin time (international normalized ratio); RBC, red blood cells; Seg, segmented neutrophil; Stab, stab neutrophil; T-bil, total bilirubin; TP, total protein; UA, uric acid; WBC, white blood cells; *γ*GTP, *γ*-glutamyl transpeptidase.

## Data Availability

The data used to support the findings of this study are included within the article.
